# Supplementary material for: Protein Secondary Structure Prediction Using Deep Convolutional Neural Fields
Source: Sci Rep. 2016 Jan 11;6:18962. doi: 10.1038/srep18962 (PMC4707437; doi:10.1038/srep18962)
Supplement: Supplementary Information [file srep18962-s1.pdf]

Supplemental file for the following manuscript

Sheng Wang, Jian Peng, Jianzhu Ma, and Jinbo Xu. Protein Secondary Structure Prediction using Deep Convolutional Neural Fields.

S.1 Training on the JPred dataset

In order to show that our method does not over-fit and to perform a comparison with JPred, we trained seven DeepCNF models using the JPred training data (http://www.compbio.dundee.ac.uk/jpred4/about.shtml), which has 1338 training proteins. We divided this dataset into 7 groups according to the JPred cross-validation sets and each time used 6 of them to train one DeepCNF model.

We use the same model architecture and hyperparameters determined by our previous method. That is, our DeepCNF model has 5 hidden layers and each layer has 100 different neurons. The window size is set to 11, and the regularization factor is set to 50.

We tested our seven DeepCNF models on the 149 JPred test proteins, which have no overlap with the JPred training data. See Supplemental Table 1 for the Q3 accuracy obtained by our models.

Supplemental Table 1. Q3 accuracy on the 149 JPred test proteins, of the 7 DeepCNF models trained by the JPred training data.

**Q3(%)**							
**1**	**2**	**3**	**4**	**5**	**6**	**7**	**Avg±std**
84.8	85.1	84.8	84.9	85.0	84.8	84.8	84.9±0.1

We also trained one DeepCNF model using the whole JPred training set and tested it on five test sets: CullPDB, CB513, CASP10, CASP11 and CAMEO. See Supplemental Table 2 for the results.

Supplemental Table 2. Q3 accuracy of the DeepCNF model trained on the whole JPred training set. Five test sets are evaluated: CullPDB, CB513, CASP10, CASP11 and CAMEO.

**Q3(%)**				
**CullPDB**	**CB513**	**CASP10**	**CASP11**	**CAMEO**
84.5	83.6	84.3	83.9	83.7

S.2 The figures comparing DeepCNF with other prediction methods on CullPDB

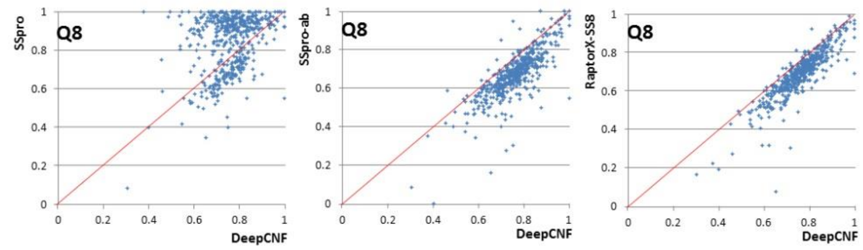

(a)

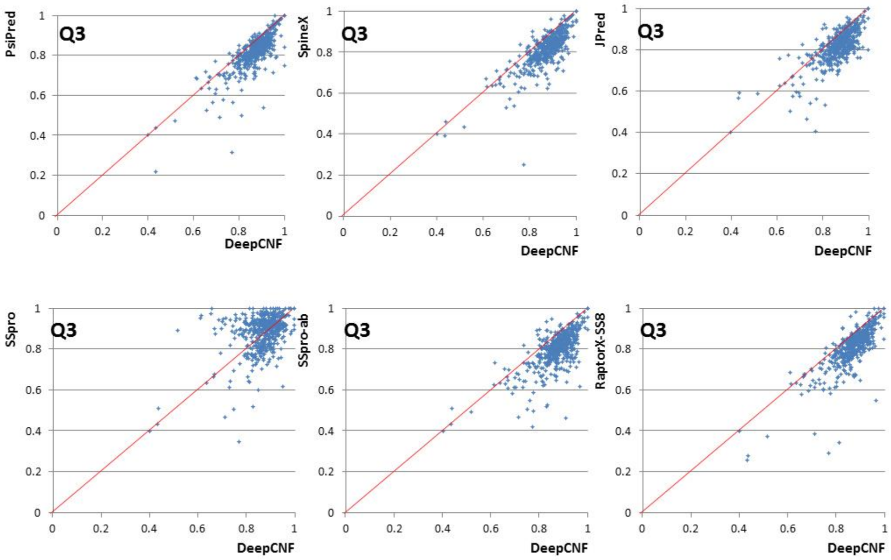

(b)

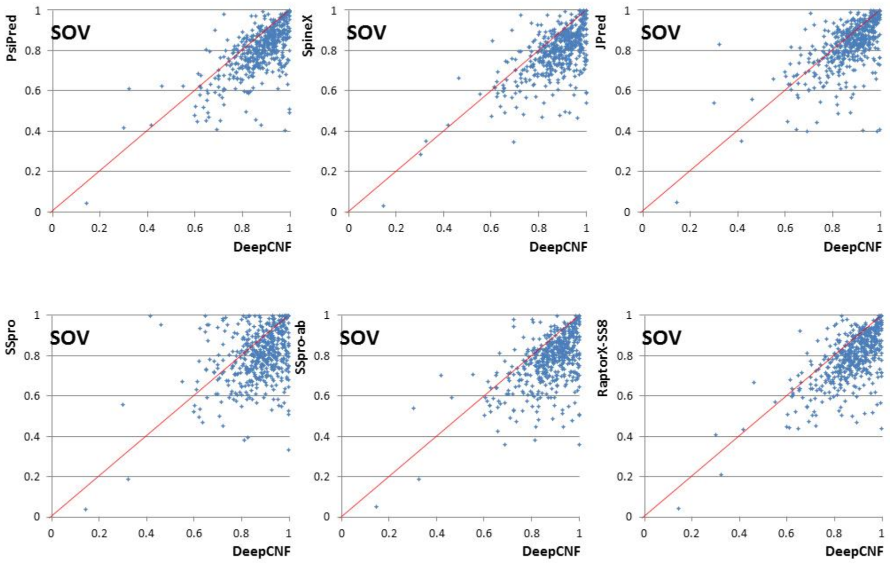

(c)

Supplemental Figure S1. (a) Head-to-head comparison of Q8 accuracy between DeepCNF and three methods: SSpro (with template), SSpro-ab (without template), and RaptorX-SS8. Head-to-head comparison of Q3 accuracy **(b)** and SOV score **(c)** between DeepCNF and the following methods: PSIPRED, SPINE-X, JPRED, SSpro (with template), SSpro-ab (without template), and RaptorX-SS8.

S.3 The figures comparing DeepCNF with other prediction methods on CB513

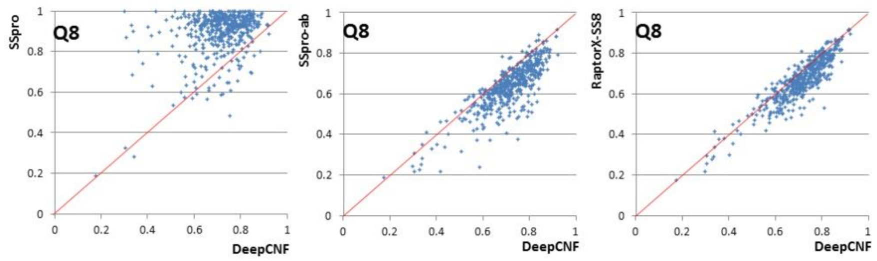

(a)

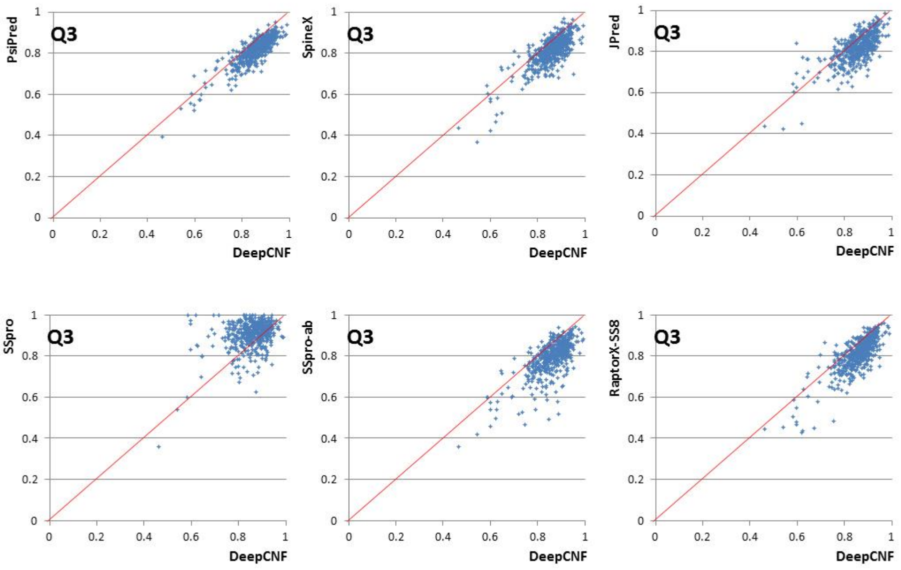

(b)

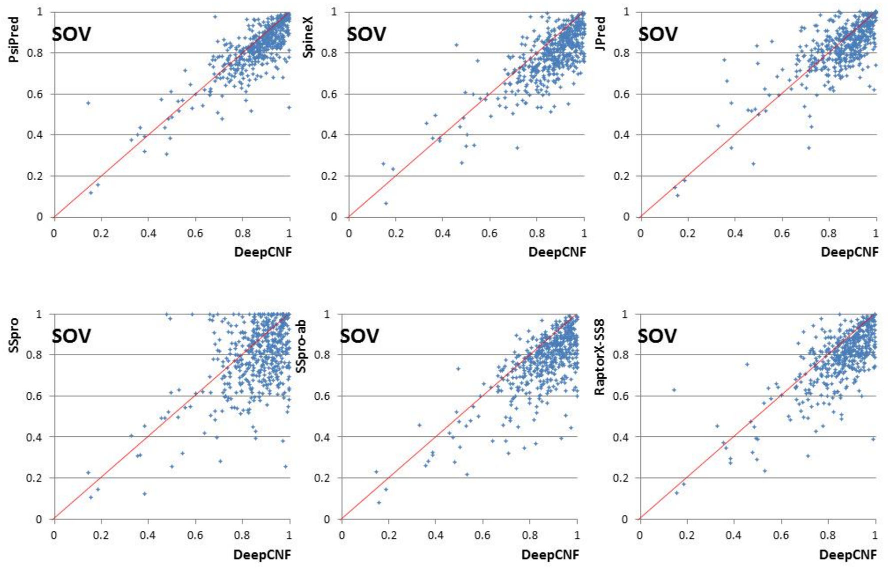

(c)

Supplemental Figure S2. (a) Head-to-head comparison of Q8 accuracy between DeepCNF and three methods: SSpro (with template), SSpro-ab (without template), and RaptorX-SS8. Head-to-head comparison of Q3 accuracy **(b)** and SOV score **(c)** between DeepCNF and the following methods: PSIPRED, SPINE-X, JPRED, SSpro (with template), SSpro-ab (without template), and RaptorX-SS8.

S.4 The figures comparing DeepCNF with other prediction methods on CASP10

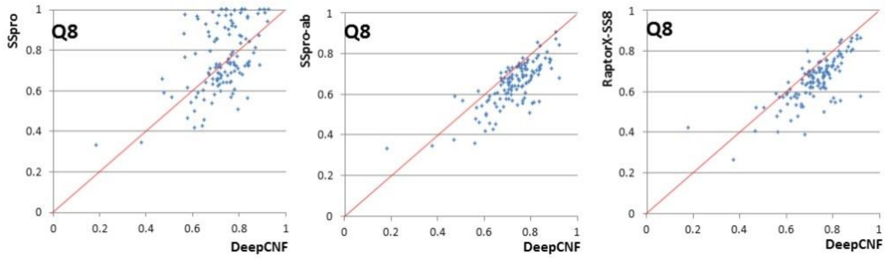

(a)

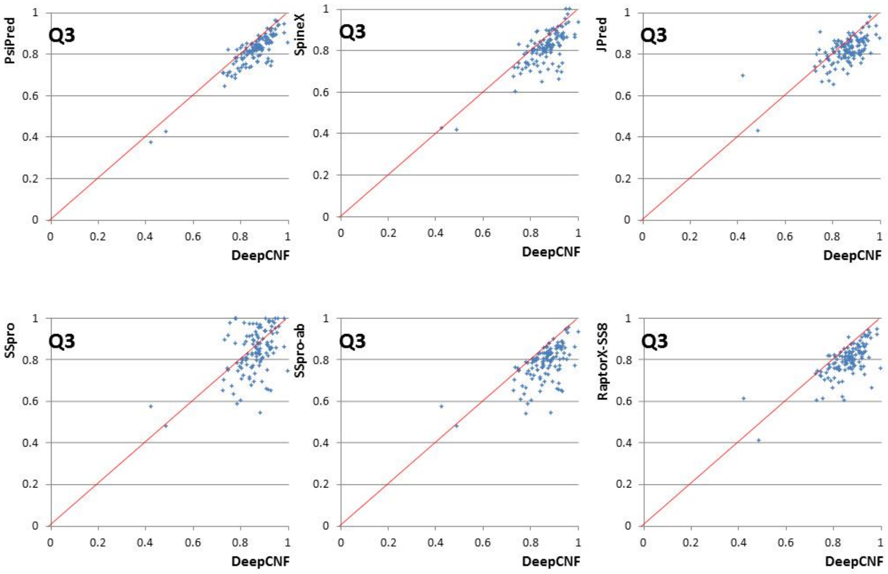

(b)

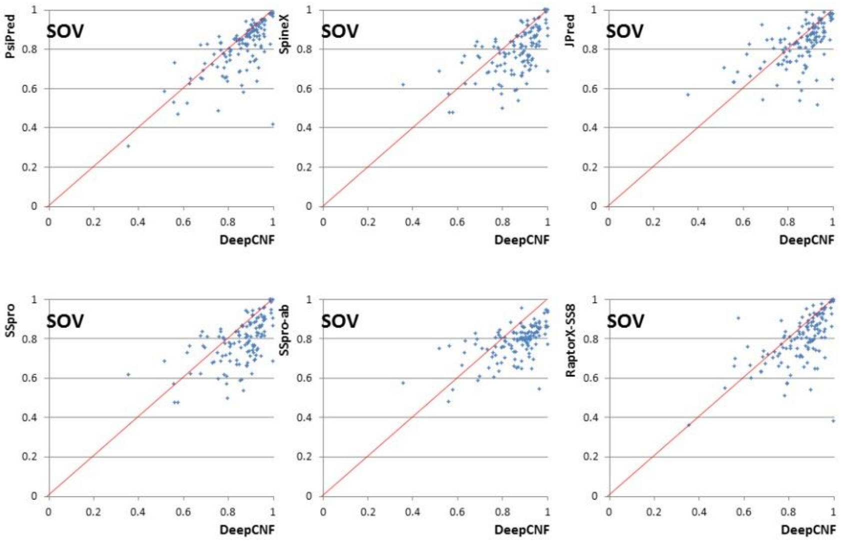

(c)

Supplemental Figure S3. (a) Head-to-head comparison of Q8 accuracy between DeepCNF and three methods: SSpro (with template), SSpro-ab (without template), and RaptorX-SS8. Head-to-head comparison of Q3 accuracy **(b)** and SOV score **(c)** between DeepCNF and the following methods: PSIPRED, SPINE-X, JPRED, SSpro (with template), SSpro-ab (without template), and RaptorX-SS8.

S.5 The figures comparing DeepCNF with other prediction methods on CASP11

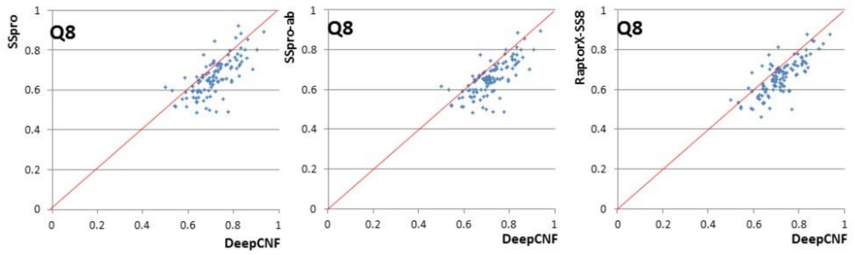

(a)

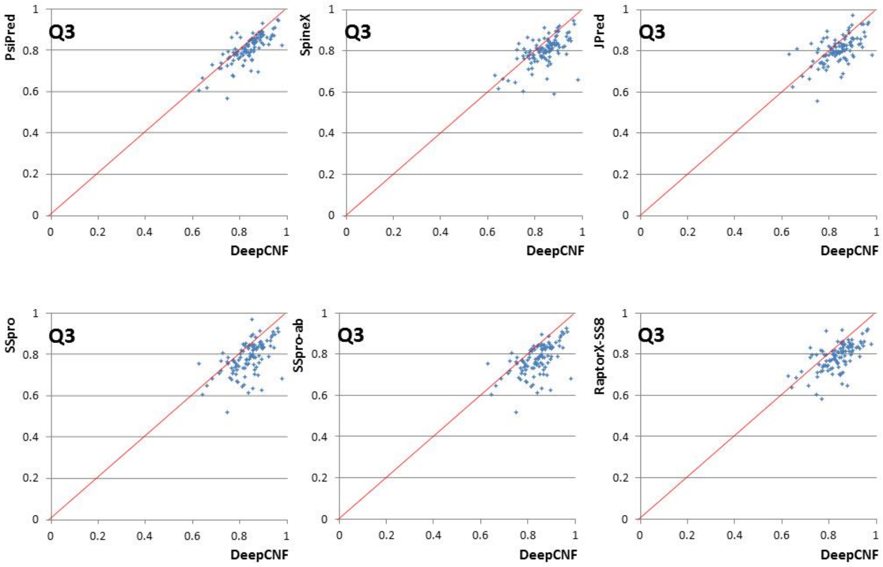

(b)

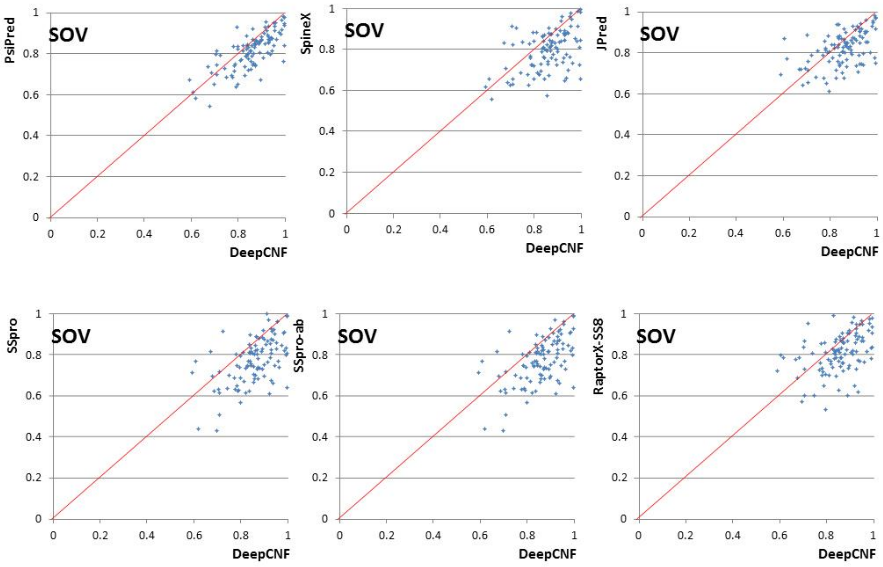

(c)

Supplemental Figure S4. (a) Head-to-head comparison of Q8 accuracy between DeepCNF and three methods: SSpro (with template), SSpro-ab (without template), and RaptorX-SS8. Head-to-head comparison of Q3 accuracy **(b)** and SOV score **(c)** between DeepCNF and the following methods: PSIPRED, SPINE-X, JPRED, SSpro (with template), SSpro-ab (without template), and RaptorX-SS8.

S.6 The figures comparing DeepCNF with other prediction methods on the CAMEO dataset

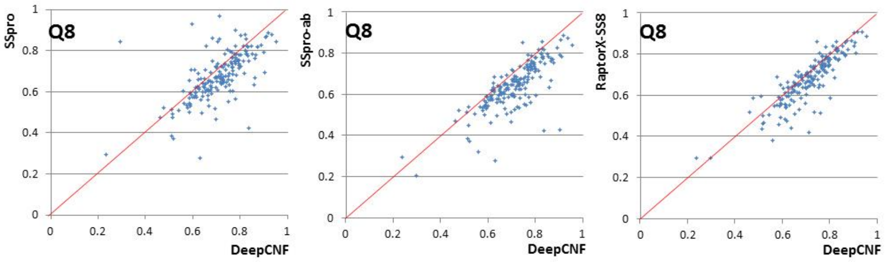

(a)

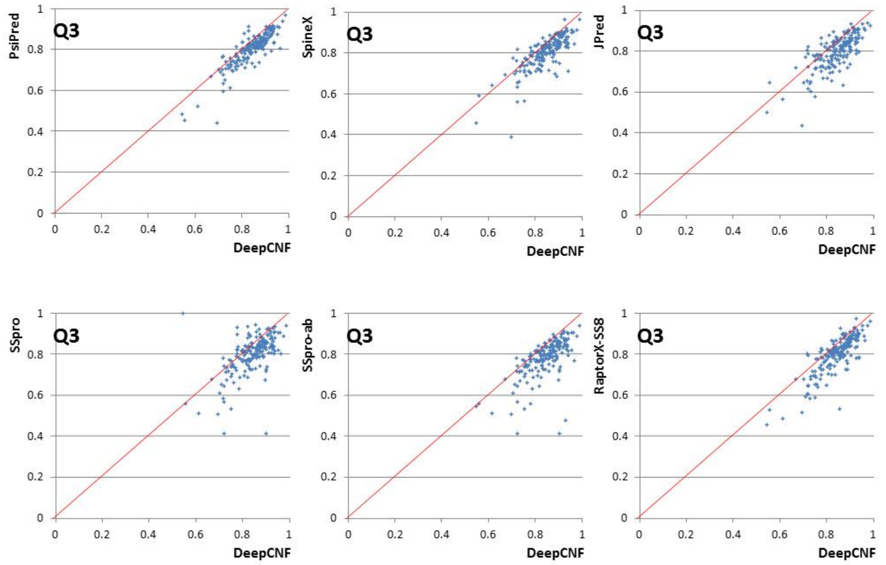

(b)

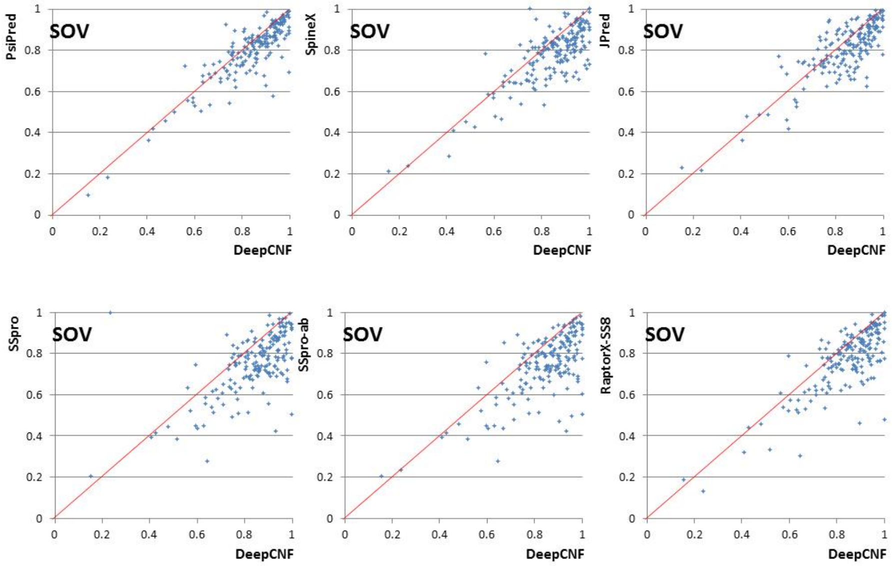

(c)

Supplemental Figure S5. (a) Head-to-head comparison of Q8 accuracy between DeepCNF and three methods: SSpro (with template), SSpro-ab (without template), and RaptorX-SS8. Head-to-head comparison of Q3 accuracy **(b)** and SOV score **(c)** between DeepCNF and the following methods: PSIPRED, SPINE-X, JPRED, SSpro (with template), SSpro-ab (without template), and RaptorX-SS8.

S.7 Statistical test of difference between DeepCNF and the other methods on four test sets

P-value. A statistical test indicates that the advantage of DeepCNF over other methods is significant, as shown in Supplemental Table S3. The p-values in the table are calculated by pairwise student's t-test between DeepCNF and the other methods. The smaller the p-value, the more significant the advantage of DeepCNF over others is. In summary, DeepCNF not only obtains better Q3 accuracy, but also better SOV score.

Supplemental Table S3. This table shows the P-values between DeepCNF and the following methods: SSpro(without template), SSpro(with template), SPINE-X, PSIPRED, JPRED and RaptorX-SS8 on five test sets in terms of Q8, Q3 accuracy, and SOV score. The smaller the p-value, the more significant the difference between two methods is.

Methods	CullPDB	CB513	CASP10	CASP11	CAMEO
**Q8 accuracy**					
SSpro (with template)	7.40E-33	1.09E-125	0.016	7.70E-11	7.13E-07
Sspro (without template)	1.65E-97	4.45E-92	1.44E-21	6.81E-17	2.15E-26
RaptorX-SS8	1.67E-73	4.88E-66	6.58E-18	3.92E-16	1.92E-11
**Q3 accuracy**					
PSIPRED	7.43E-42	1.07E-29	7.99E-13	2.65E-06	1.55E-15
SPINE-X	3.67E-79	4.2E-72	1.34E-15	8.61E-09	1.65E-21
JPRED	1.92E-53	1.48E-48	4.36E-12	2.50E-07	5.09E-22
SSpro (with template)	1.84E-04	1.67E-37	0.062	3.85E-12	3.01E-11
Sspro (without template)	3.36E-80	1.77E-76	2.69E-20	2.36E-15	2.72E-23
RaptorX-SS8	1.51E-47	1.19E-36	4.54E-14	9.03E-11	9.03E-16
**SOV score**					
PSIPRED	1.27E-50	2.19E-26	1.32E-05	2.50E-06	1.14E-09
SPINE-X	8.70E-61	9.27E-54	2.04E-11	4.37E-10	4.68E-18
JPRED	3.24E-21	2.04E-09	4.99E-03	1.82E-05	3.21E-09
SSpro (with template)	1.53E-29	2.37E-20	2.04E-11	3.31E-14	1.21E-18
Sspro (without template)	6.10E-64	4.49E-59	1.40E-13	5.47E-15	1.55E-23
RaptorX-SS8	1.14E-33	5.28E-38	6.74E-07	6.68E-06	9.62E-15

S.8 Segment OVerlap (SOV) Score

The Segment Overlap score (SOV) measures overlap between the observed and the predicted secondary structure segments instead of per-residue accuracy, proposed by Zemla et. al. (Zemla, Venclovas, Fidelis, & Rost, 1999). The predictions that have high per-residue accuracy but deviate from experimental segment length distributions have lower SOV scores (Im, 2008). SOV score ranges from 0 to 1 with 1 indicating the perfect overlap.

Brief description of SOV from (Zemla, et al., 1999) is as follows. To calculate SOV, the (predicted) secondary structure of one protein sequence is parsed into segments such that each segment has a single secondary structure type. Let S1 be the observed secondary structure and S2 the predicted secondary structure. For each type $i \in \{H, E, C\}$, $S(i)$ is the set of segment pair $(s1, s2)$ with type i where s1 is from S1, s2 is from S2 and $s1$ and $s2$ overlap in at least one residue. That is, $S(i) = \{(s1, s2) : s1 \cap s2 \neq 0 \text{ and } s1\ and\ s2 \text{ have type } i\}$. In contrast, $S'(i) = \{s1 : s1 \cap s2 = 0 \text{ and } s1\ and\ s2 \text{ have type } i\}$.

Then the segment overlap score between S1 and S2 is calculated as follows.

$$SOV(S1, S2) = \frac{1}{N} \sum_{i \in \{H,E,C\}} \sum_{(s1,s2) \in S(i)} \frac{\min(s1,s2) + \sigma(s1,s2)}{\max(s1,s2)} \cdot l(s1) \quad (1)$$

where $\min(s1, s2)$ is the length of the overlap between s1 and s2, $\max(s1, s2)$ is the length of the total span of s1 and s2, and $l(s1)$ is the length of s1, $\sigma(s1, s2)$ is defined as min($\max(s1, s2) - \min(s1, s2)$, $\min(s1, s2)$, $\left\lfloor \frac{l(s2)}{2} \right\rfloor$, $\left\lfloor \frac{l(s1)}{2} \right\rfloor$) , and N is defined as $\sum_{i \in \{H,E,C\}} N(i)$ where $N(i) = \sum_{(s1,s2) \in S(i)} l(s1) + \sum_{s1 \in S'(i)} l(s1)$

References

Im, I. G. (2008). *Predicting Protein Secondary Structure Using Markov Chain Monte-Carlo Simulation*: ProQuest.

Zemla, A., Venclovas, Č., Fidelis, K., & Rost, B. (1999). A modified definition of Sov, a segment‐based measure for protein secondary structure prediction assessment. *Proteins: Structure, Function, and Bioinformatics, 34*(2), 220-223.
